# Supplementary material for: Examining the mental health outcomes of school-based peer-led interventions on young people: A scoping review of range and a systematic review of effectiveness
Source: PLoS One. 2021 Apr 15;16(4):e0249553. doi: 10.1371/journal.pone.0249553 (PMC8049263; doi:10.1371/journal.pone.0249553)
Supplement: S1 Appendix — (DOCX) [file pone.0249553.s002.docx]

# S1 Appendix

## Example systematic review search (PsycINFO)

| 1 | exp mental health/ or psychological well-being/ |
| --- | --- |
| 2 | exp child psychiatry/ or exp child psychology/ |
| 3 | exp mental disease/ or anxiety disorder/ or autism/ or emotional disorder/ or mental instability/ or mood disorder/ or personality disorder/ or psychosis/ or thought disorder/ or exp mental disease assessment/ |
| 4 | (Mental Health or Psych* or Wellbeing or Well-being or Mental Disorder* or Mental Illness or Psychological Issue* or Emotional Wellbeing or Bully* or Psychiatric Disorder* or Marginalised Group* or Substance Misuse or Substance Abuse or Addict* or Drug Abuse or Drug Misuse or Alcohol Abuse or Alcohol Misuse or Aggress* or Suicid*5 or Suicidal Ideation or Self Harm or Self-Harm or Self Destructive Behaviour or Self-Destructive Behaviour or Self-Injurious Behaviour or Self Injurious Behaviour or Non-Suicidal Self-Injury or Non Suicidal Self Injury or Self-Inflicted Wound* or Self Inflicted Wound* or Self Mutilation or Self-Mutilation or Self Poisoning or Self-Poisoning or Attempted Suicide or Trauma* or Depress* or Anxi* or Low Mood or Eating Disorder or School Transition or LGBTQ*).mp. [mp=title, abstract, heading word, table of contents, key concepts, original title, tests & measures] |
| 5 | 1 or 2 or 3 or 4 |
| 6 | exp peer counseling/ or exp peer group/ |
| 7 | exp friend/ |
| 8 | student/ or elementary student/ or high school student/ or middle school student/ |
| 9 | exp friendship/ |
| 10 | (Peer Support or Peer Buddying or Peer Counsel* or Peer Intervention* or Peer Listening or Befriending or Peer* or Peer Relation* or Friend* or Peer Support Group* or Peer-led or Cross-age or Cross age or Peer to Peer or Peer-to-Peer or Paraprofessional or Para-professional or Peer-delivered or Peer Delivered or Peer Education).mp. [mp=title, abstract, heading word, table of contents, key concepts, original title, tests & measures] |
| 11 | 6 or 7 or 8 or 9 or 10 |
| 12 | exp school/ or high school/ or kindergarten/ or middle school/ or primary school/ |
| 13 | education/ or education program/ or health education/ or interdisciplinary education/ or learning environment/ or mentoring/ or "outcome of education"/ or school attendance/ or student retention/ |
| 14 | exp mentor/ |
| 15 | (School* or Primary School* or Secondary School* or Educational Setting* or College* or Sixth Form* or Elementary School* or High School* or Junior School* or Special Education*).mp. [mp=title, abstract, heading word, table of contents, key concepts, original title, tests & measures] |
| 16 | 12 or 13 or 14 or 15 |
| 17 | exp school health service/ |
| 18 | exp intervention study/ |
| 19 | (Intervention or Train* or Program* or School Based Intervention* or Group Intervention* or Scheme* or Initiative* or Approach*).mp. [mp=title, abstract, heading word, table of contents, key concepts, original title, tests & measures] |
| 20 | 16 or 17 or 18 |
| 21 | 5 and 11 and 16 and 20 |
| 22 | limit 21 to (human and (preschool child <1 to 6 years> or school child <7 to 12 years> or adolescent <13 to 17 years>)) |
