# Supplementary material for: Examining the mental health outcomes of school-based peer-led interventions on young people: A scoping review of range and a systematic review of effectiveness
Source: PLoS One. 2021 Apr 15;16(4):e0249553. doi: 10.1371/journal.pone.0249553 (PMC8049263; doi:10.1371/journal.pone.0249553)
Supplement: S2 Appendix — (DOCX) [file pone.0249553.s003.docx]

## S2 Appendix

## Risk of bias assessments

Table A.1 Risk of bias assessments of randomised controlled trials (RCTs) using Cochrane Risk of Bias Assessment Tool

| Author and year | Random sequence generation | Allocation concealment | Selective outcome reporting | Performance bias | Detection bias | Attrition bias | Other bias |
| --- | --- | --- | --- | --- | --- | --- | --- |
| *Mason-Jones et al (2013)* | x | x | x | ? | x | x | x |
| *Sebire et al. (2018)* | ✓ | ✓ | ✓ | ✓ | ✓ | ✓ | ✓ |
| *Shah et al. (2000)* | ✓ | ✓ | ✓ | ✓ | x | ✓ | x |
| *Wyman et al. (2010)* | ✓ | ✓ | ✓ | ? | ✓ | ✓ | ✓ |

Table A.2 Risk of bias assessments of quasi-experimental studies

| Author and year | Cause and effect clear | Similar comparison group | Comparison receiving similar care | Presence of control group | Multiple measurements of outcome | Follow-up complete | Comparisons measured in same way | Outcomes measured in reliable way | Appropriate statistical analysis |
| --- | --- | --- | --- | --- | --- | --- | --- | --- | --- |
| *Ellis (2004)* | ✓ | ✓ | ? | ✓ | ✓ | ✓ | ✓ | ✓ | ✓ |
| *Ellis (2009)* | ✓ | ✓ | ✓ | ✓ | ✓ | ? | ✓ | ✓ | ✓ |
| *Froh (2004)* | ✓ | ✓ | ✓ | ✓ | ✓ | ✓ | ✓ | x | ✓ |
| *Roach (2014)* | ✓ | ✓ | x | ✓ | ✓ | x | ✓ | ? | ✓ |
| *Song et al. (2018)* | ✓ | ✓ | ? | ✓ | ✓ | x | ✓ | ✓ | ✓ |
| *Yogev and Ronen (1982)* | ✓ | ✓ | x | ✓ | ✓ | x | ✓ | ✓ | ✓ |

Table A.3 Risk of bias assessments of studies with a pre- and post-test design

| Author and year | Clear study question | Clear eligibility criteria | Representative sample | All eligible participants enrolled | Sufficient sample size | Intervention clearly described | Outcomes measures defined and reliable | Blind assessments | Loss to follow-up under 20% and accounted for | Statistical tests suitable | Outcome measures recorded at multiple points | Statistical analysis at group level determines group effects |
| --- | --- | --- | --- | --- | --- | --- | --- | --- | --- | --- | --- | --- |
| *Bausano (2006)* | ✓ | ✓ | X | X | N/A | ✓ | ✓ | N/A | X | ✓ | X | X |
