# Supplementary material for: Examining the mental health outcomes of school-based peer-led interventions on young people: A scoping review of range and a systematic review of effectiveness
Source: PLoS One. 2021 Apr 15;16(4):e0249553. doi: 10.1371/journal.pone.0249553 (PMC8049263; doi:10.1371/journal.pone.0249553)
Supplement: Attachment — Submitted filename: PLOS One Peer interventions.docx [file pone.0249553.s004.docx]

## The focus of this review is on the range and effectiveness of peer led programs. Many schools use peer approaches informally to help with transitions, i.e., assign a “buddy” to help a new student acclimating to a new school. This makes practical sense and can bridge a stressful transition and build a sense of social connectedness which is important to student mental health in general.

## Peers may be in a better position to identify students struggling with mental health problems and other challenges than adults in the schools. It makes practical sense to provide some mental health education to all students in schools so they are able to identify a student in crisis and know what to do to facilitate that student being connected to an adult who can get help them the help they need. There are established effective models for this (e.g., Teen Mental Health First Aid).

It was impressive that 11 databases were used (very comprehensive) but, to me, this review missed the mark. A great deal of time and effort went into this review **but it is not clear to me which programs are effective and can be scaled up**. Maybe that was because the studies are not adequate to really say anything conclusive about the range and effectiveness of peer led programs. If there are not any, please be explicit about this.

It would be great if you could better synthesize/summarize the details for the **training** provided for peer leaders – how many hours, what is the training content, were adults involved? In an ideal world, peer leaders should be trained to inform a trusted adult in the school about students who are at risk. The **role** of the peer leaders should also be more explicit, please synthesize and summarize this as well. It would have been ideal for the authors to describe the **role** the peers played and elements of the interventions that have a positive impact on outcomes. Importantly, were the peer leaders connected with adults or supervised by adults? **The Sources of Strength program is very good but I believe 10% off the staff are trained in addition to the students and there are adults that supervise the student leaders and support them. This is a robust model that has been around for decades and has been broadly disseminated.**  Maybe this should be outlined in the results and discussion sections with subheadings (Training of peer, role of peer, school adult involvement).

Which components or elements of programs appear to be effective and**, more importantly,** which appear to be harmful? One of the most important take-aways for me is the authors pointing out the risk of harm to students or iatrogenic effects. These should be clearly outlined, this point should be explicit so that schools can use this in decision-making and either prepare for this to avoid these outcomes or decide to select an adult-led program. Guilt was mentioned as an outcome. Did peer leaders feel guilty because they were not able to prevent bullying or suicide, and that is why they feel guilty? If so, this is a major issue that should be elucidated for schools.

Given that there are several adult-led school-based programs that are effective (e.g, Youth Aware of Mental Health, Good Behavior Game, Teen Mental Health Frist Aid) , perhaps the recommendation should be that these effective programs should be prioritized over the peer-led programs until a stronger evidence base is established for peer programs.

## It does not seem that these programs prevented bullying, suicidal behaviors or the outcomes they targeted but they appear to make students feel more connected to one another and increase self-esteem or the peer leaders. Perhaps the follow-up of the studies was not long enough to see impacts. Perhaps the samples size was too small? Perhaps the schools did not properly measure these outcomes. The authors need to be more explicit about what is needed to move the science forward in this area. As it appears that is 62% of schools in England use these programs, there needs to be a push for evaluation of the efforts.

Also, the sustainability of these programs is also of interest. Are there any data on these programs being sustained long-term?
